# Dynamic biochemical tissue analysis detects functional selectin ligands on human cancer tissues

**DOI:** 10.1038/s41598-019-44838-4

**Published:** 2019-06-11

**Authors:** Eric W. Martin, Ramiro Malgor, Vicente A. Resto, Douglas J. Goetz, Monica M. Burdick

**Affiliations:** 1 Biomedical Engineering Program, Russ College of Engineering and Technology, Athens, USA; 2 Department of Chemical and Biomolecular Engineering, Russ College of Engineering and Technology, Athens, USA; 3 Department of Biomedical Sciences, Heritage College of Osteopathic Medicine, Athens, USA; 4 Edison Biotechnology Institute, Ohio University, Athens, OH 45701 USA; 5 Department of Otolaryngology, University of Texas-Medical Branch, Galveston, TX 77555 USA

**Keywords:** Biophysical methods, Biomedical engineering

## Abstract

Cell adhesion mediated by selectins (expressed by activated endothelium, activated platelets, and leukocytes) binding to their resepective selectin ligands (expressed by cancer cells) may be involved in metastasis. Therefore, methods of characterizing selectin ligands expressed on human tissue may serve as valuable assays. Presented herein is an innovative method for detecting functional selectin ligands expressed on human tissue that uses a dynamic approach, which allows for control over the force applied to the bonds between the probe and target molecules. This new method of tissue interrogation, known as dynamic biochemical tissue analysis (DBTA), involves the perfusion of molecular probe-coated microspheres over tissues. DBTA using selectin-coated probes is able to detect functional selectin ligands expressed on tissue from multiple cancer types at both primary and metastatic sites.

## Introduction

Accruing evidence suggests metastasis is facilitated by adhesive interactions between E-, P-, and L-selectin (expressed by activated vascular endothelium, activated vascular endothelium and activated platelets, and leukocytes, respectively) and their respective ligands (expressed by cancer cells)^1–16^. For instance, P-selectin knockout mice exhibit significantly lower rates of metastasis compared to wild type controls in colon carcinoma models^1,2^, and selectin inhibition through heparin treatment decreases metastasis in mice^3–5^. Additionally, human head and neck cancer cells can express L-selectin ligands that are capable of binding to L-selectin under low, lymphatic flow conditions^6^. More recently, E-selectin-mediated interactions have been shown to be involved in the homing of circulating breast cancer cells expressing E-selectin ligands to bone marrow expressing E-selectin^7^. Yet, despite these findings, characterization of functional selectin ligands on human tissue remains elusive, perhaps due to the nature of the bonds formed by selectins with their ligands (i.e., selectin/selectin-ligand bonds).

Work in the field of cell adhesion has revealed the unique force-lifetime relationship of selectin/selectin-ligand bonds^17–30^. L-selectin requires a minimum threshold shear in order to mediate adhesion^26^, and known selectin ligands demonstrate differential binding activities to selectins under shear stress^31^. Marshall *et al*. used bond lifetime distribution analysis from atomic force microscopy experiments coupled with flow chamber experiments to show that the bond lifetime of the P-selectin/PSGL-1 complex initially increases, reaches a maximum, and then decreases with increasing amounts of applied force^27^. This phenomenon is known as the ‘catch to slip’ bond transition and helped provide an explanation for the shear threshold effect^26,29^.

Because of the unique force-lifetime relationship of selectin/selectin-ligand bonds, molecules that can bind to selectins under static conditions should be distinguished from functional selectin ligands that interact with selectins in a dynamic environment^32^. Consequently, much of the *in vitro* evidence implicating selectins and their ligands in metastasis comes from the adhesion analysis of human cancer cell lines to purified selectins or transfected cell-expressed selectins in flow-based assays that emulate the hemodynamic shear generated by flowing blood^12–16,33–35^. Therefore, the characterization of functional selectin ligands expressed *in situ* on tissue should also incorporate a similar element of applied force, e.g., physiologically-relevant shear stress.

Although the methodology for functionally analyzing selectin ligands expressed on cells in suspension is well-established^12–15,17,20,33–37^, techniques for detecting functional selectin ligands expressed *in situ* on tissue have not been developed. Previously, Stamper-Woodruff^38^ and modified Stamper-Woodruff^39^ assays have been utilized, but these assays do not incorporate the continuous application of well-defined, physiologically-relevant wall shear stress. To date, the best practice has been immunostaining with either selectin chimeras^40^ or antibodies that recognize critical components of selectin ligands, e.g., sialofucosylated moieties, expressed on tissue^41–44^. However, immunostaining is a static biochemical tissue analysis (SBTA) that is unable to ascertain if a potential selectin ligand is able to mediate (rolling) adhesion under conditions with continuously applied wall shear stress. As a result, the relevance of functional selectin ligand expression by human cancerous tissue, distinct from circulating tumor cells, as a biomarker is not well understood.

To fill this gap, we have developed a flow-based assay, termed dynamic biochemical tissue analysis (DBTA), to characterize the expression of functional selectin ligands expressed on tissue^45^. DBTA involves perfusing particles coated with a molecular probe (e.g., selectin-coated microspheres) over tissues of interest (e.g., expressing putative selectin ligands). By emulating the conditions in which adhesive interactions occur, the DBTA technique allows for the discovery of functional selectin ligands that are capable of mediating adhesion under flow.

Carlson *et al*. introduced DBTA with L-selectin DBTA probes using colorectal cancer as an investigational substrate^45^. The work herein extends the prior investigation by comprehensively including data for DBTA probes conjugated with antibodies against sialofucosylated structures (HECA-452, KM231, and CSLEX-1), as well as other selectins (human E- and P-selectin, and murine E-selectin). Furthermore, this work probes for glycoconjugate epitopes of purported selectin ligands in tandem with DBTA, characterizes ligands using receptor-ligand off rates, and includes an exploratory investigation of tissues from multiple organ-specific cancers at both primary and metastatic sites using DBTA.

## Results

### DBTA probe adhesion is specific, quantifiable, and discernible from immunostaining (i.e., SBTA)

**Figure 1:**
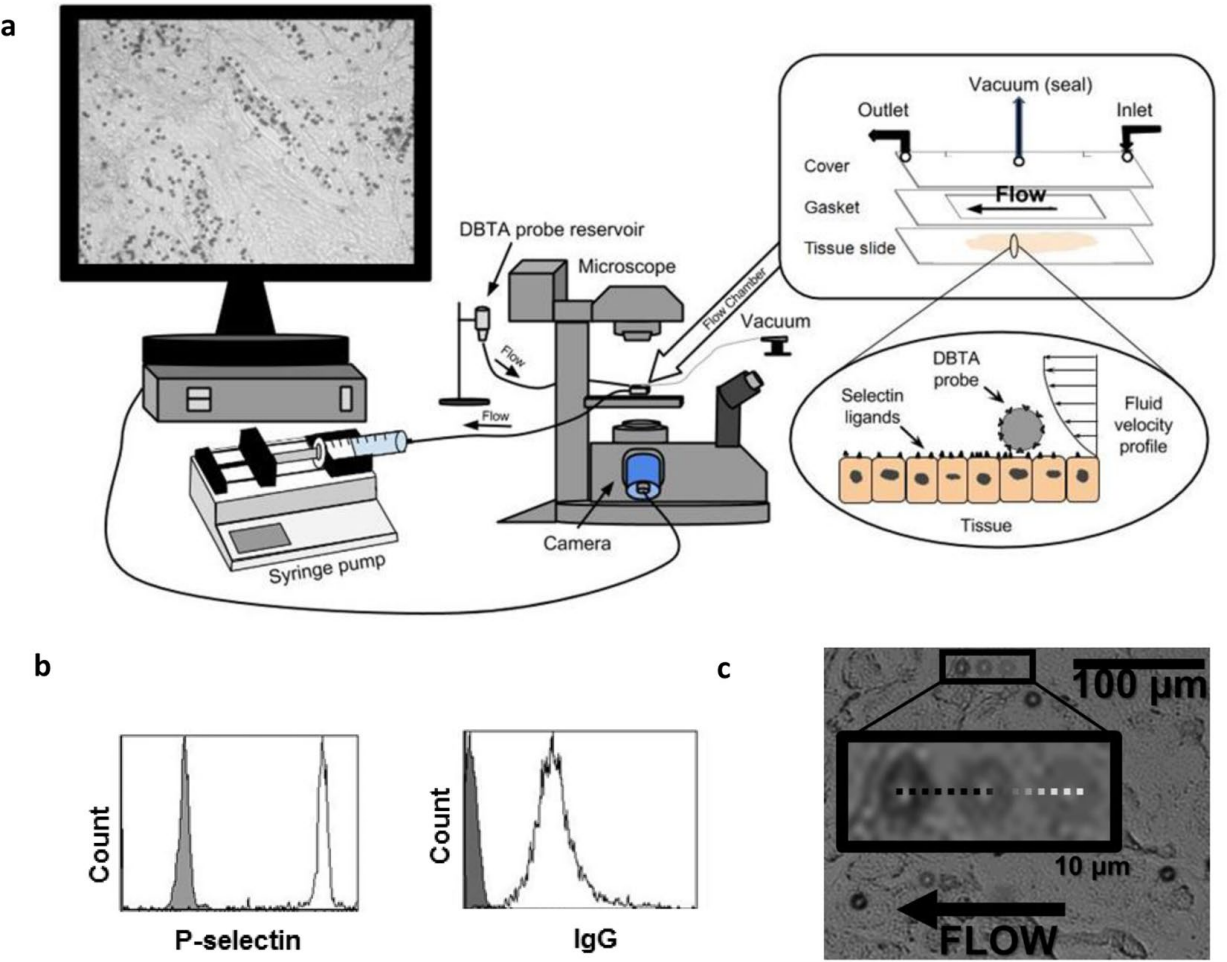
Introduction to the DBTA (dynamic biochemical tissue analysis) (**a**) DBTA probes (microspheres conjugated with a molecular probe such as a selectin or antibody) are perfused over tissue at defined wall shear stresses using a syringe pump and parallel plate flow chamber. Schematics are not to scale. (**b**) DBTA probe conjugation with the desired molecular probe (e.g., P-selectin or human IgG) can be verified with flow cytometry. (**c**) Images from three time points have been combined to show a sample P-selectin DBTA probe rolling on a colon carcinoma tissue.

Successful DBTA probe conjugation was verified via flow cytometry prior to conducting DBTA (Fig. 1 and Supplementary Figs S1 and S2). A representative DBTA probe rolling on a tissue is shown in Fig. 1c. To determine if DBTA probe adhesion is an artifact of the formalin fixation, paraffin embedding (FFPE) process, model colon and breast cancer cell lines that express functional selectin ligands^16,34,46,47^ were used to study the effect of the FFPE process on selectin ligands. It was found that the FFPE process did not increase selectin-mediated DBTA probe adhesion to these cell lines nor mock tissues consisting of sectioned cell line plugs (Supplementary Fig. S3 and our prior work^45^).

**Figure 2:**
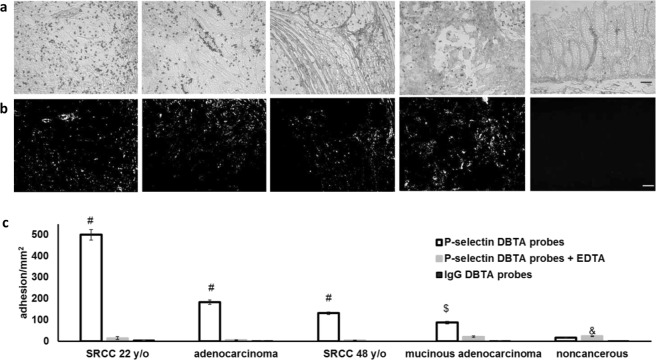
DBTA signal is specific, quantifiable, and discernible from SBTA. (**a**) P-selectin DBTA probes adhesion at 0.50 dyne/cm^2^ to colon tissue (from left to right: SRCC T4N1M0 22 y/o, adenocarcinoma T4N0M0, SRCC T4N1M0 48 y/o, mucinous adenocarcinoma T4N2M0, noncancerous colon tissue). DBTA probes appear as black circles. (**b**) P-selectin SBTA conducted on serial sections of the tissues examined in (**a**) with DBTA revealed detection of ligands that are not in complete agreement with the purported functional ligands detected via DBTA (**a**). Order of tissues is the same as (**a**). (**c**) P-selectin DBTA probe adhesion to colon tissue was specific and significantly greater than control probes. DBTA probes were perfused at 250,000 probes/mL and 0.50 dyne/cm^2^. Specificity of interaction was confirmed using 10 mM EDTA (divalent cation chelator, Ca^2+^ is required for selectin/selectin-ligand binding) and hIgG DBTA probes as negative controls. Data shown are mean adhesion ± SD of three technical replicates and are representative of independent experiments conducted on tissue sections from >10 independent cases of colon cancer and >3 independent noncancerous cases. ^#^P < 0.001, ^$^P < 0.01, and ^&^P < 0.05, compared to all others intragroup. Scale bar = 100 µm.

Since substantial *in vivo* and *in vitro* data suggesting selectin-ligand interactions may be involved in metastasis come from the P-selectin colon carcinoma models^1–3,5,12–14^, DBTA was initially performed using P-selectin DBTA probes with colon tissue as the investigational substrate (Fig. 2). P-selectin DBTA probes specifically adhered to four sample cancer tissue sections at 0.50 dyne/cm^2^ (Fig. 2 and Supplementary Videos S1–S7). The corresponding adhesion values are displayed in Fig. 2c (the left to right order of the tissues is the same). Specificity of P-selectin DBTA probe interaction with purported selectin ligands expressed by the tissue was validated using 10 mM EDTA (divalent cation chelator; Ca^2+^ is required for selectin/selectin-ligand binding) and human IgG (hIgG) DBTA probes as controls. For the noncancerous samples investigated in this study with DBTA, the adhesion of P-selectin DBTA probes to noncancerous colon tissue was not statistically different than the adhesion of control DBTA probes (e.g., background binding of hIgG DBTA probes, Fig. 2c). Tissue sections serially adjacent to the DBTA sections underwent P-selectin SBTA (immunostaining, Fig. 2b). The purported ligands detected by the static method (SBTA, Fig. 2b) were not in full spatial agreement with the purported ligands detected by the dynamic method (DBTA, Fig. 2a and Supplementary Videos S1, S4 and S7), indicating the molecular probe, P-selectin, may detect a different set of selectin ligands under shear stress.

### DBTA probe adhesion to colon carcinoma tissue is force-dependent

**Figure 3:**
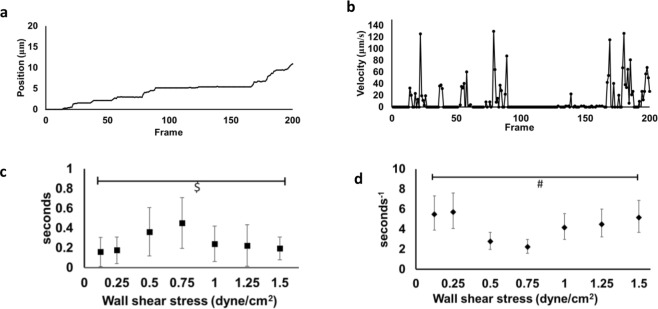
DBTA probe adhesion to signet ring cell colon carcinoma (SRCC) tissue is force-dependent. **(a)** Position profile of a P-selectin DBTA probe rolling on a tissue section (SRCC 48 y/o case) at 0.75 dyne/cm^2^. Video frame rate = 175 fps (Supplementary Video S8). **(b)** Velocity profile of the P-selectin DBTA probe tracked in (**a**). (**c**) Increasing levels of applied force initially increased then decreased the lifetime of P-selectin DBTA probe adhesion to SRCC tissue. Data shown are mean adhesion duration in seconds ± SD of 30 separate pauses at each wall shear stress collected in a 100 μm × 100 μm region. ^$^F = 12.59 > F_0.01, 6,273_ = 2.80 **(d**) Increasing levels of applied force initially decreased then increased the off-rate (k_off_) of P-selectin DBTA probe from the SRCC tissue. Data shown represent the estimation of P-selectin DBTA probe k_off_ using the method of maximum likelihood^48^ from 30 separate pauses at each wall shear stress. Error bars represent 95% CI. ^#^Levene’s test = 4.0818 (equivalent to P = 0.0014).

The position and velocity of a representative P-selectin DBTA probe as it rolled over a signet ring cell carcinoma (SRCC 48 y/o) tissue sample is shown in Fig. 3a,b, respectively (Supplementary Video S8). This type of tracking data was used to determine DBTA probe adhesion lifetimes and to estimate receptor-ligand disassociation parameters^48^ that were calculated from 30 pause time measurements collected at each shear stress increment over the range 0.125–1.50 dyne/cm^2^. These values are reported as average DBTA probe pause time in Fig. 3c and are in the range of 0.1–0.5 seconds. Figure 3d provides maximum likelihood estimation of the receptor-ligand disassociation parameter^48^, k_off_, for each incremental shear stress, and are within the range of 2–6 inverse seconds. Regardless of distribution treatment, it was found that both the average pause duration and maximum likelihood estimator for k_off_ indicate that P-selectin DBTA probe adhesion (pauses) to the putative selectin ligands expressed by the tissue are force-dependent, consistent with expected selectin/selectin-ligand bond behavior^27–30^.

### The presence of the HECA-452 epitope does not dictate a functional P-selectin ligand

**Figure 4:**
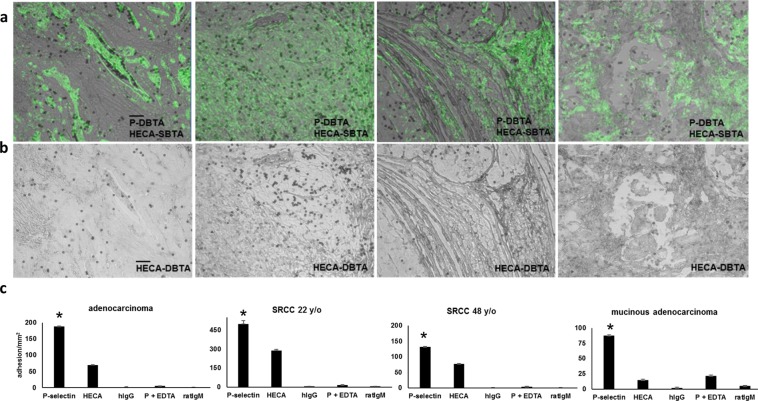
The presence of sLeX/A does not dictate a functional P-selectin ligand. (**a**) SBTA with the HECA-452 monoclonal antibody (green pseudocolor), which detects both sLeX and sLeA, has been combined with an image showing the adhesion patterns of P-selectin DBTA probes (Fig. 2). HECA-452 SBTA was conducted on serial sections. For the adenocarcinoma and SRCC 22 y/o tissues, but not the SRCC 48 y/o and mucinous adenocarcinoma tissues, there was an agreement between P-selectin DBTA and SBTA with HECA-452 in which the regions of tissue that express sLeX or sLeA mediate adhesion to P-selectin DBTA probes. However, with regard to the SRCC 48 y/o and mucinous adenocarcinoma tissues, not all regions that express sLeX or sLeA, as detected by HECA-452 SBTA, appeared to mediate binding to P-selectin DBTA probes (Supplementary Videos S1, S4, and S7). **(b)** HECA-452 DBTA probes (microspheres coated with HECA-452) adhered to tissue at 0.50 dyne/cm^2^ (Supplementary Videos S9 and S10). **(c)** The greatest amount of adhesion to the tissue occurred with the P-selectin DBTA probes. Probes were perfused at 250,000 probes/mL and 0.50 dyne/cm^2^. Specificity of interaction was validated using hIgG DBTA probes, 10 mM EDTA, and rIgM DBTA probes as controls. Data shown are mean adhesion ± SD of three technical replicates and are representative of independent experiments conducted on tissue sections from >3 independent cases of colon cancer. *P < 0.0025, compared to all others intragroup. Scale bar = 100 µm.

Because known selectin ligands are glycoconjugates (e.g., proteins, peptides, and lipids conjugated with terminal sialofucosylated moieties)^49,50^, the expression of sLeX or sLeA as a necessary requirement for a putative P-selectin ligand to mediate adhesion with P-selectin DBTA probes under wall shear stress was explored. SBTA (immunostaining) with HECA-452 monoclonal antibody that recognizes primarily sLeX/A was conducted on serial sections (Fig. 4). The signal from SBTA with HECA-452 is shown as green pseudocolor and has been combined with an image showing the P-selectin DBTA probe adhesion pattern(s) for reference. Interestingly, it was observed that P-selectin DBTA probes adhered in regions not detected by HECA-452 SBTA, and not all regions that expressed sLeX or sLeA, as detected by HECA-452 SBTA, appeared to mediate binding to P-selectin DBTA (Supplementary Videos S1, S4 and S7).

Subsequently, due to the well-documented force dependency of selectin ligands, DBTA was conducted with HECA-452 DBTA probes to study the effect of applied force on the detection capabilities of this antibody (Fig. 4b). Specificity of HECA-452 DBTA probe interaction was validated using rat IgM isotype control coated DBTA probes. Analysis of the adhesion values of the four colon cancer tissues indicates the HECA-452 DBTA probes underwent specific adhesion, but this interaction was observed in fewer regions of interest and with significantly lower amounts of adhesion than the P-selectin DBTA probes (Fig. 4c). Although the HECA-452 DBTA probes adhered to these tissues, it was also found that the probes did not interact with all regions of tissue that displayed adhesion with P-selectin DBTA probes, nor did they adhere to all areas detected by SBTA with HECA-452 (Fig. 4a,b, and Supplementary Videos S1, S4, S9 and S10). Taken together, these results imply that a functional selectin ligand is not equivalent to the presence of the HECA-452 epitope, and that expression of the HECA-452 epitope is not a necessary requirement.

### P-selectin DBTA probe adhesion in regions not detected by SBTA

**Figure 5:**
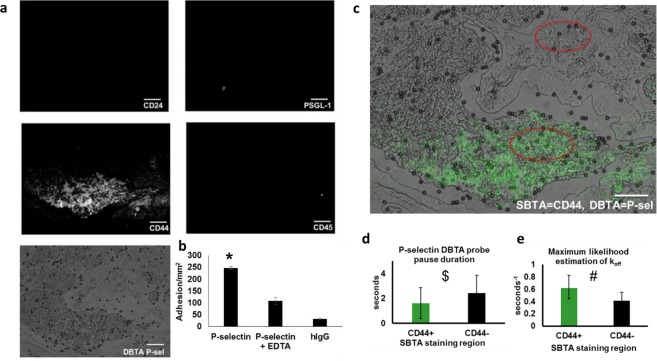
Adhesion in regions not detected by SBTA implies the presence of potentially distinct P-selectin ligands. (**a**) In the same regions of tissue that displayed P-selectin DBTA probe adhesion (Supplementary Video S11), examination of serial sections with SBTA (immunostaining) revealed no detectable levels of CD24 or PSGL-1 epitopes. CD44 expression was detected, but not all regions displaying specific reactivity with the P-selectin DBTA probes used in DBTA were recognized with the CD44 antibody in SBTA. Follow-up CD45 SBTA ruled out the possibility of DBTA probe interaction with infiltrated leukocytes, in agreement with the lack of PSGL-1 detection. (**b**) P-selectin DBTA probes specifically adhered to the tissue, with respect to the negative controls. Data shown are mean adhesion ± SD of three technical replicates and are representative of independent experiments conducted on tissue sections from >3 independent cases of colon cancer. *P < 0.0025 compared to all other conditions. (**c**) A direct comparison using a composite image of P-selectin DBTA probe adhesion (microspheres) and SBTA detection of CD44 (green pseudocolor) on colon adenocarcinoma tissue. Red ellipses indicate regions analyzed in (**d**,**e**). **(d)** Characterization of the adhesion of P-selectin DBTA probes to functional selectin ligands that were and were not detected with SBTA using CD44 antibody in (**a**). Data shown are mean pause duration ± SD of 30 separate pauses (n = 30). ^$^P = 0.0068. (**e**) Data shown represent the estimation of P-selectin DBTA probe k_off_ using the method of maximum likelihood from 30 separate pauses (n = 30). Error bars represent 95% CI. ^#^Mann-Whitney U-value = 1152.5 (equivalent to P < 0.001 for t-test for normal distribution). All probes were perfused at 500,000 probes/mL and 0.50 dyne/cm^2^. Specificity of interaction was confirmed using 10 mM EDTA and hIgG DBTA probes as negative controls. Scale bar = 100 µm. These results imply the presence of potentially distinct P-selectin ligands in the tissue sections.

Serial tissue sections were immunostained with antibodies that recognize the protein structures of known P-selectin ligands, CD24 and CD44 (identified using cancer cell lines^13–15^), and directly compared to regions displaying P-selectin DBTA probe reactivity (Fig. 5 and Supplementary Video S11). Specificity of P-selectin DBTA probe interaction was validated using 10 mM EDTA and hIgG DBTA probes as negative controls. Immunostaining for the leukocyte marker, CD45, was conducted to determine if DBTA probes were adhering to ligands (i.e., PSGL-1) expressed on the membrane of infiltrated leukocytes. Epitopes for CD24, CD45, and PSGL-1 were not detected (Fig. 5a). A significant amount of CD44 expression was detected, but not all regions displaying specific reactivity with the P-selectin DBTA probes were recognized with SBTA using the CD44 antibody (Fig. 5a–c). These findings may indicate the presence of a selectin ligand(s) other than the well-reported P-selectin ligands that are expressed by cancer cells.

To further probe the identity of the unknown selectin ligand(s) using the dynamic method, pause time distribution analysis was conducted in the regions of colon adenocarcinoma with CD44 expression and compared to regions without CD44 expression as determined using SBTA. Figure 5c shows the region of colon adenocarcinoma tissue that displayed P-selectin ligands detected using DBTA combined with green pseudocolor representing the CD44 detected via SBTA (immunostaining). The red ellipses (100 μm × 150 μm) indicate the regions in which pause time distribution analysis was conducted. Statistically different pause time distributions were observed for the respective CD44+ and CD44− regions (Fig. 5c–e). Combined with the findings from CD44 SBTA, these data suggest DBTA can potentially distinguish between distinct functional P-selectin ligands expressed *in situ* on colon carcinoma tissue.

### Functional P-selectin ligands are expressed on colon, lung, ovarian, pancreatic, and stomach cancer tissues

**Figure 6:**
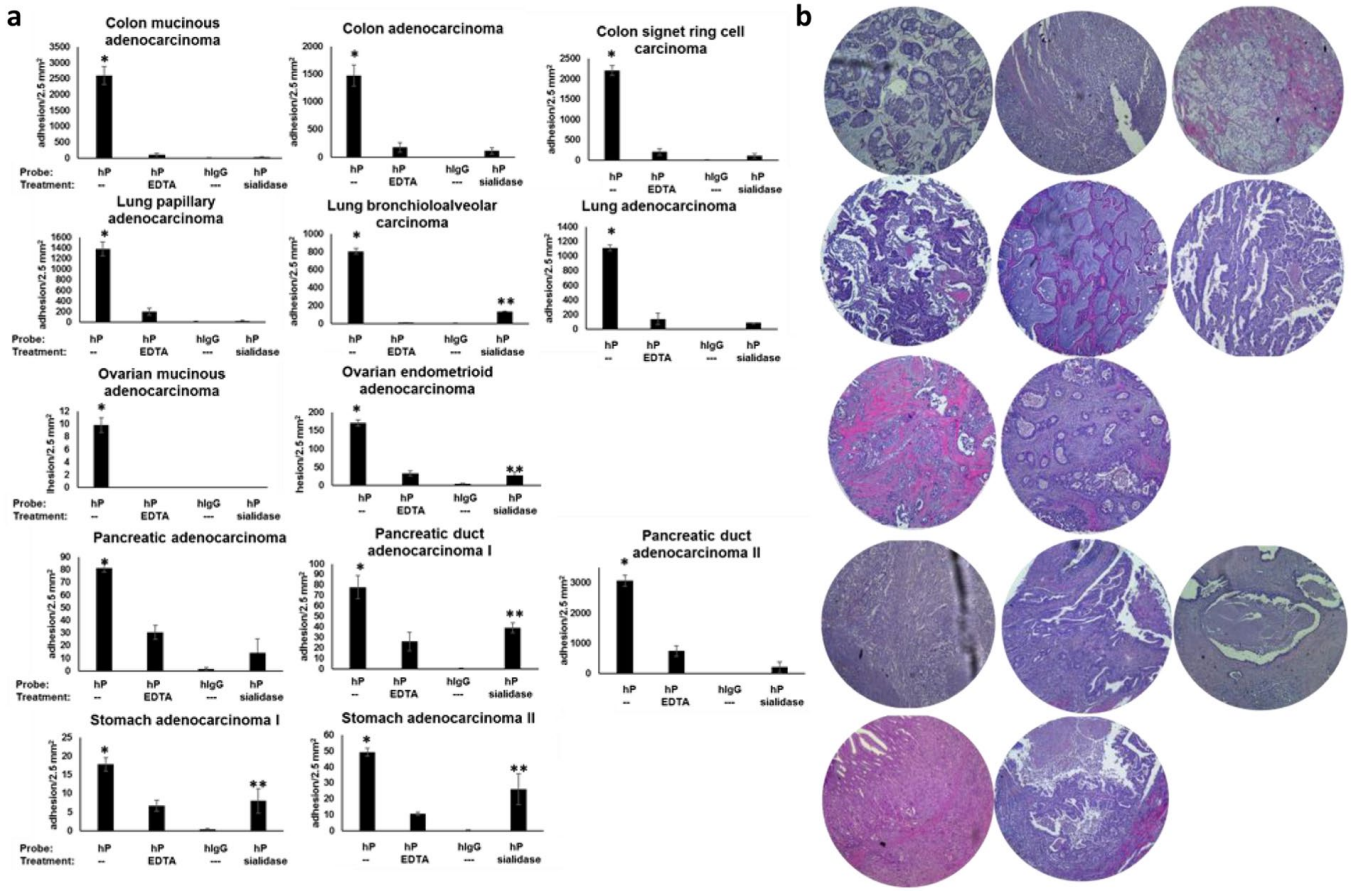
P-selectin DBTA probes specifically adhere to consecutive serial sections of colon, lung, ovarian, pancreatic, and stomach cancer tissues. (**a**) Functional P-selectin ligands are expressed on colon, lung, ovarian, pancreatic, and stomach cancer tissues. DBTA probes were perfused over three serial sections at 0.50 dyne/cm^2^ and 500,000 probes/mL. Specificity of interaction was confirmed using 10 mM EDTA, hIgG DBTA probes, and human P-selectin (hP) DBTA probes perfused over sialidase-treated tissue as negative controls. Data shown are mean adhesion ± SEM of three technical replicates from each of three independent serial sections. * and **P < 0.05 for P-selectin (hP) with respect to the three controls (hP to hP + EDTA, hIgG, and hP + sialidase) and the sialidase-treated tissue with respect to hIgG and hP + EDTA, respectively. See Supplementary Table S1 for P-values and Supplementary Videos S12–S17 for adhesion patterns. (**b**) Corresponding H&E images on the right are shown in the same order as (**a**). Core diameter is 2.5 mm.

DBTA with P-selectin probes was used to investigate additional types of cancer tissue for the expression of functional P-selectin ligands. The data reported in Fig. 6 represent the mean adhesion values of P-selectin DBTA probes to three consecutive serial sections for each individual cancer case. P-selectin DBTA probe adhesion to these tissue samples was identified as specific with respect to the negative controls, including sialidase treatment that cleaves sialic acids traditionally rendering functional activity to selectin ligands, indicating these tissues express ligands that can bind to P-selectin under flow (Fig. 6 and Supplementary Videos S12–S17).

### DBTA detects functional selectin ligands expressed on tissue from multiple solid tumors at both primary and metastatic sites

**Figure 7:**
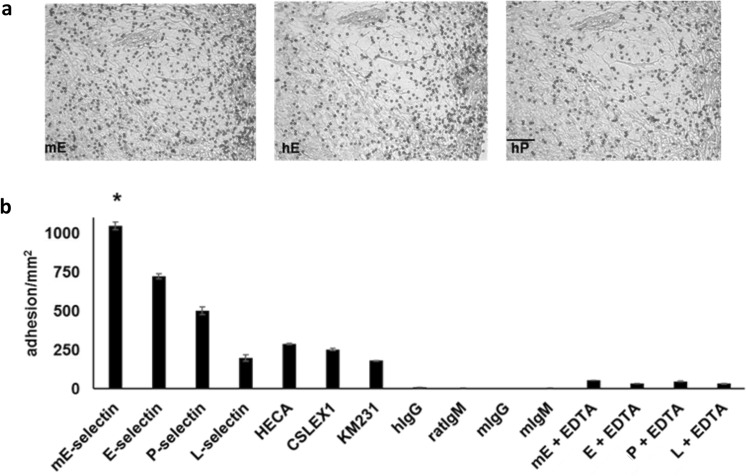
The greatest amount of specific adhesion to SRCC occurs with the mE-selectin DBTA probe. mE-, E-, P-, L-selectin, HECA-452, CLSEX-1, and KM231 DBTA probes adhered to SRCC 22 y/o tissue. **(a)** Representative images of the adhesion patterns for mE-, E-, and P-selectin are shown. (**b**) The greatest amount of specific DBTA probe adhesion occurred with the mE-selectin probe. Probes were perfused at 250,000 probes/mL and 0.50 dyne/cm^2^. Specificity of interaction was confirmed using 10 mM EDTA (divalent cation chelator, Ca^2+^ is required for selectin/selectin-ligand binding) as well as with hIgG, rat IgM, mouse IgG, and mouse IgM DBTA probes as negative controls. Data shown are mean adhesion ± SD of three technical replicates and are representative of independent experiments conducted on tissue sections from >3 independent cases colon cancer. Scale bar = 100 µm. *P < 0.001 compared to all other conditions. See Supplementary Fig. S2 for validation of DBTA probe conjugation. Scale bar = 100 µm.

Evidence from the adhesion literature suggests that the criteria for a given moiety to serve as a ligand for E-selectin is generally not as stringent as that for the other selectins^46,51,52^. Hence, we speculated that probes conjugated with E-selectin would have a higher level of binding, compared to probes conjugated with other selectins (P- and L-selectin) and antibodies for sLeX/A (HECA-452 for sLeX and sLeA, CSLEX-1 for sLeX, and KM231 for sLeA). Herewith, DBTA probes with distinct molecular conjugations were separately perfused over n >3 independent cases of colon cancer. These probes were conjugated using equimolar incubation concentrations and were perfused at the same shear stress. The greatest amount of specific adhesion was found to occur with the murine E-selectin (mE) DBTA probe (Fig. 7). In further experiments conducted on lung cancer tissues using mE-, E-, and P-selectin DBTA probes, it was also found that the greatest amount of specific adhesion occurred with the mE-selectin DBTA probe (Supplementary Fig. S4).

**Figure 8:**
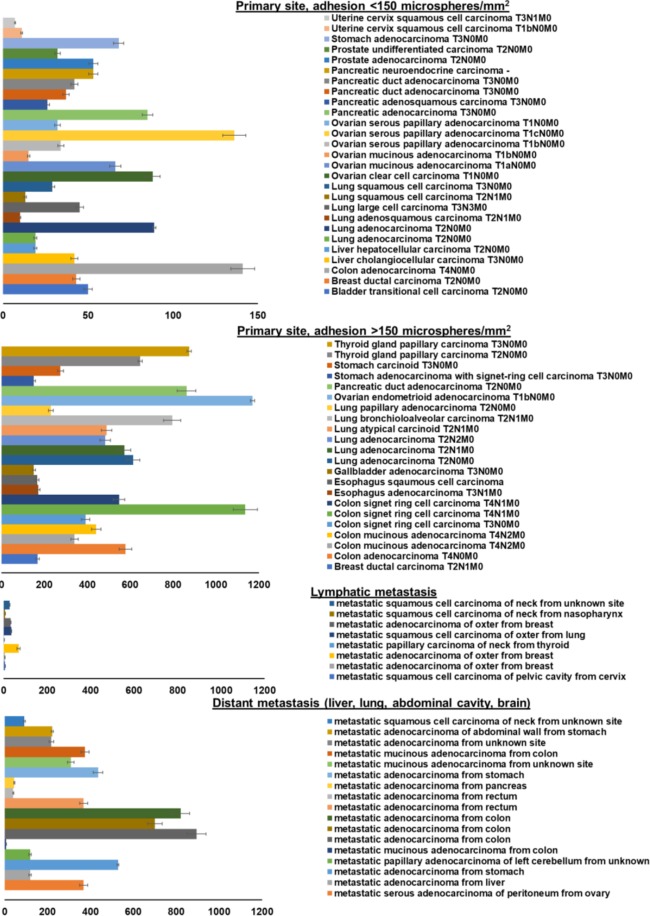
DBTA detects functional selectin ligands expressed on tissue from multiple solid tumors at both primary and metastatic sites. mE-selectin DBTA probes adhesion to cancerous tissues derived from primary and metastatic sites. DBTA probes were perfused at 500,000 probes/mL and 0.50 dyne/cm^2^. Data shown are mean adhesion per mm^2^ ± SD of three technical replicates and include the deduction of control probe adhesion (viz. mean of P-selectin adhesion minus the greater mean value of either P-selectin + EDTA or IgG control probes).

Since the intent of this study was to detect the larger set of putative functional selectin ligands, a probe with high analytical sensitivity and specificity was desired to conduct a large-scale assessment of multiple tissues. Thus, the mE-selectin DBTA probe was used in the analysis of greater than 150 cases of cancer to screen for the expression of functional selectin ligands (Fig. 8). Overall, probe adhesion was observed on 76 of the 165 cases examined with adhesion levels ranging from 7 to 1170 specifically adherent mE-selectin DBTA probes/mm^2^ on uterine cervix squamous cell carcinoma and ovarian endometrioid adenocarcinoma, respectively, indicating broad levels of functional selectin ligand expression (Fig. 8 and Supplementary Table S2). Additionally, although the data are exploratory, the levels of adhesion on tissues from metastatic sites also seemed to be dependent on the location with observed adhesion greater on samples from distant sites compared to lymphatic sites (Supplementary Fig. S5).

## Discussion

Prior to the development of DBTA, the traditional research tools used to investigate selectin ligands expressed on tissue were ill-equipped to assess functionality. These traditional methods, namely SBTA immunostaining, rely on probing tissue with antibodies or selectins, and do not simulate the shear stress conditions under which selectins can functionally bind their ligands. This inability to ascertain the functionality of selectin ligands expressed on tissue is the main limitation of traditional methods, and is further compounded by the assertation that selectin ligands must be expressed with the right attributes, e.g., post-translational modifications (Supplementary Fig. S6), to be functional^32^. DBTA circumvents these limitations, in part, by incorporating the element of continuous, physiologically-relevant wall shear stress that provides the ability to specifically detect and ascertain the functionality of selectin ligands expressed on tissue.

Traditional SBTA immunostaining methods have heavily relied on detection of glycoconjugate structures with monoclonal antibodies for either the protein or the carbohydrate domains, rather than probing with selectins. One target of immunostaining is CD24, which acts as a P-selectin ligand on breast cancer cells^15^, and another is the set of well-known functional P-selectin ligands expressed by colon cancer, which are the multiple variant forms of CD44 (e.g., HCELL)^13,14,53^. These variants of CD44 contain selectin-binding domains in the form of O-linked glycans, with sialofucosylated structures (e.g., sLeX/A), that are covalent modifications of the peptide backbone^13,14^. Monoclonal antibody HECA-452 primarily recognizes two critical moieties expressed by selectin ligands, sLeX/A^41,42^. However, nonsialylated selectin ligands have been shown to exist^54^, and the HECA-452 antibody only recognizes the presence of sLeX/A without detecting any of the other post-translational modifications, such as sulfation, that may be required to mediate adhesion between selectins and their associated ligands^55^. Additionally, there is evidence that HECA-452 is non-function blocking antibody for sLeX, indicating that HECA-452 recognition does not dictate full functionality^56^. In Fig. 4, there is some agreement between P-selectin DBTA and SBTA with HECA-452, in which the regions of tissue that express sLeX or sLeA can mediate adhesion. However, not all regions positive in HECA-452 SBTA appear to mediate binding to P-selectin DBTA probes, and more importantly, not all regions that can mediate adhesion were detected with SBTA using HECA-452. Taken together, these data could imply the existence of a set of functional selectin ligands that do not express sLeX/A^13,33^.

To account for the effect of applied force in the detection of sLeX/A, HECA-452 DBTA was conducted, resulting in observable, specific adhesion to the tissue surface but with an apparent reduction of detection capability with respect to HECA-452 SBTA. This finding is exemplified in Fig. 4: P-selectin DBTA probes globally adhered to the tissue (SRCC 22 y/o) within the examination window while SBTA of the tissue with HECA-452 revealed widespread signal, but HECA-452 DBTA probes only bound to a subset of the same examination window. This result of diminished HECA-452 DBTA detection capability is perhaps attributable to the typical nature of the antibody-antigen bond, which primarily exhibits a decrease in bond lifetime with increasing force, rather than an initial increase then decrease in bond lifetime with increasing force that is a hallmark of the selectin/selectin-ligand catch-slip disassociation pathway^27–29,57^.

More generally, this study illustrates the challenge in detecting functional selectin ligands expressed *in situ* on tissue using traditional methods, i.e., SBTA with selectins. In Figs 2 and 4, there is evidence that suggests the same molecular probe can generate contrary signals when used in the two different settings of DBTA and SBTA (immunostaining). In Fig. 4 the adhesion patterns of HECA-452 DBTA probes did not appear to adhere to all regions positive in SBTA with HECA-452, while P-selectin DBTA probes adhered to more regions than what would have been predicted based on the signal from SBTA with P-selectin. This result is perhaps attributable to the inherent, unique force-dependent nature of the bonds formed between selectins and their ligands^15,27–29,57–60^ and is our rationale for using selectin DBTA probes with emulated hemodynamic flow to detect functional selectin ligands expressed *in situ* on tissue.

Both the average pause duration and maximum likelihood estimator for k_off_ shown in Fig. 3 reveal that P-selectin DBTA probe adhesion (pauses) to the purported selectin ligands expressed by the tissue is force-dependent, regardless of distribution treatment. All types of bonds are force-dependent, that is the associated lifetimes can change with applied force. The lifetime of the bond between the selectin on the microsphere and putative selectin ligand(s) on the tissue initially seems to increase with applied force, before decreasing, data that have been shown with other force-lifetime measurements (e.g., AFM) of selectin/selectin-ligand bonds^27^. The k_off_ values experimentally calculated with DBTA using pause time distribution analysis in this work, which range from 0.6–6 inverse seconds, are in agreement with other reports that investigated stressed off-rates for P-selectin/P-selectin ligand complexes^15,27,57–61^.

As can be seen in Fig. 7, and supported by other work, there exists a complicated intersection and overlap of functional E-, P-, and L- selectin ligands^2,13,32,46,49,51,53^. Because of this overlap and the desire to screen with high analytical sensitivity and specificity, mE-selectin was used to investigate numerous tissue samples for the expression of functional selectin ligands. Despite being murine derived, mE-selectin is 73% similar to human E-selectin, but mE-selectin has been shown to display higher affinity than its human counterpart for certain structures^62^. Therefore, to investigate the prevalence of functional selectin ligand expression on cancer tissue, 165 cases of various solid tumors at primary and metastatic sites were investigated using DBTA with murine E-selectin. Figure 8 shows that specific mE-selectin DBTA probe adhesion occurred on multiple cases of breast, colon, esophagus, liver, lung, ovarian, pancreatic, prostate, stomach, thyroid, and uterine cervix cancers, demonstrating the presence of functional selectin ligands. In addition, probe adhesion occurred on multiple cases of breast, cervical, colon, liver, lung, ovarian, pancreatic, rectal, and stomach cancer tissues sampled from metastatic sites. In total, tissues from 165 cases of cancer were examined, of which mE-selectin DBTA probe adhesion was observed on 76 (Supplementary Fig. S7 and Table S2). To the best of our knowledge, the work herein is the first report of widespread functional selectin ligand expression *in situ* by multiple solid tumors as detected by a flow-based, functional assay.

Although there is substantial data that implicate selectin ligands in metastasis^1–5,12,13,15,16,63^, a majority of which is from functional analysis of cell line models, selectin inhibition, and knockout experiments, studies investigating the expression of functional selectin ligands *in situ* on tissue are lacking. Detection and characterization of functional E-, P-, L- selectin ligands expressed *in situ* on tissue may prove worthwhile as the ability of the primary tumor to express functional selectin ligands may be related to the ability of a circulating tumor cell to express functional selectin ligands. With the corroborating evidence from Fig. 8, it could be hypothesized that a hypothetical CTC arising from a tissue expressing selectin ligands may be able to retain selectin ligand expression and interact at distant sites that express selectins, such as activated endothelium which expresses E- and P-selectin, platelets that express P-selectin, or leukocytes that express L-selectin.

In summary, DBTA using selectin-coated probes is able to detect functional selectin ligands expressed on tissue from multiple cancer types at both primary and metastatic sites. The characterization of functional selectin ligands on human tissue no longer remains elusive, and such methods could be incorporated into novel multiplexed tissue interrogation assays. More broadly, characterization of functional adhesion molecules (e.g., selectin ligands, integrins, etc.) expressed on human tissue may serve as valuable diagnostic and prognostic assays.

## Methods

### Ethics

Anonymous, de-identified tissue samples purchased from US Biomax, Inc. (Rockville, MD) were classified as IRB exempt by Ohio University’s Office of Research Compliance.

### Antibodies and selectins

Recombinant human E-selectin-hIgG1 Fc chimera, recombinant human P-selectin-hIgG1 Fc chimera, recombinant human L-selectin-hIgG1 Fc chimera and recombinant mouse E-selectin-hIgG1 Fc chimera were purchased from R&D Systems (Minneapolis, MN). Human IgG1 isotype control was purchased from Sigma-Aldrich (St. Louis, MO). Purified rat anti-human cutaneous lymphocyte antigen [HECA-452, primarily recognizes sialyl Lewis X (sLeX) and sialyl Lewis A (sLeA)], mouse anti-human CD15s (CSLEX-1) [recognizes sialyl Lewis X (sLeX)], rat IgM isotype, mouse IgM isotype, phycoerythrin (PE)-conjugated mouse anti-human CD62E (E-selectin), PE-conjugated mouse anti-human CD62P (P-selectin), PE-conjugated mouse anti-human CD62L (L-selectin), PE-conjugated mouse IgG1 isotype control, PE-conjugated rat anti-mouse CD62E (mouse E-selectin), PE-conjugated rat IgG2a isotype control, mouse anti-human PSGL-1 (KPL-1), mouse anti-human CD24 (ML5), mouse anti-human CD45 (HI30), mouse IgG1 isotype, mouse IgG2a isotype, and rat IgG2a isotype were purchased from BD Biosciences (San Jose, CA). Mouse anti-sialyl Lewis A (KM231) was purchased from EMD Millipore (Billerica, MA). Rat anti-human CD44 (HERMES-1) was purchased from Thermo Fisher Pierce Biotechnology (Waltham, MA). Allophycocyanin (APC)-conjugated F(ab’)_2_ goat anti-human IgG was purchased from Jackson ImmunoResearch (Westgrove, PA). Alexa Fluor 647 conjugated mouse anti-human IgG (H + L) polyclonal antibody, Alexa Fluor 647 conjugated goat anti-mouse IgG (H + L) polyclonal antibody, Alexa Fluor 647 conjugated goat anti-rat IgM polyclonal antibody, Alexa Fluor 647 conjugated goat anti-human IgG (H + L) polyclonal antibody, Alexa Fluor 647 conjugated goat anti-rat IgG (H + L) polyclonal antibody, and Alexa Fluor 568 conjugated goat anti-mouse IgM polyclonal antibody were obtained from Life Technologies (Carlsbad, CA). Supplemental Information Antibodies: Alexa Fluor 488 conjugated goat anti-mouse IgG polyclonal antibody, Alexa Fluor 488 conjugated goat anti-mouse IgM, Alexa Fluor 488 goat anti-Rat IgM were obtained from Life Technologies.

### DBTA probe preparation

Polystyrene microspheres from Bangs Laboratories (Fishers, IN) with a mean diameter of 10 μm were conjugated using a technique previously described^51^. In brief, microspheres were washed in TBS pH = 4.0, twice in DPBS, and then incubated at 2.5 × 10^7^ microspheres/mL in the desired molecular probe in DPBS at equimolar concentrations (relative to 10 ug/mL of recombinant human P-selectin Fc chimera). Conjugated microspheres were washed then blocked with 1% BSA,1% FBS in DPBS. Immediately prior to conducting DBTA, microsphere conjugation was verified via flow cytometry where 100,000 microsphere samples were incubated with the appropriate fluorophore-conjugated antibody or control (e.g., PE-conjugated mouse anti-human CD62P or PE-conjugated mouse IgG1) at 5 µg/mL concentration in 0.1% BSA, DPBS for 30 min. These microspheres were washed twice with 1% BSA and once in DPBS then resuspended in DPBS and analyzed by a FACSAria Special Order Research Product flow cytometer/sorter (BD Biosciences, San Joes, CA). Prior to perfusion in the dynamic biochemical tissue analysis, DBTA probes (conjugated microspheres) were resuspended, unless noted otherwise, at a concentration of 5 × 10^5^ probes/mL in DPBS and verified with a Scepter 2.0 handheld, automated cell counter (EMD Millipore).

### Tissue preparation

Deidentified, formalin-fixed paraffin-embedded (FFPE) human tissue slides in single tissue format or in microarray format (US Biomax, Rockville, MD)^64^ were deparaffinized by heating at 60 °C for 90 min followed by serially incubating with xylene, 100% ethanol, 95% ethanol, 70% ethanol, and DPBS^65^. Prior to immunostaining for peptide/protein structures, tissues underwent heat-activated epitope retrieval at 95 °C for 25 minutes in 10 mM sodium citrate, 0.05% Tween 20 at pH of 6.0; which was immediately followed by a wash in DPBS. To cleave terminal sialic acid residues that are prominent selectin ligand glycotopes, treatment with sialidase^66^ (also known as neuraminidase) was conducted using 0.1 U/ml *Vibrio cholerae* neuraminidase (Roche Biochemicals, Indianapolis, IN) in DPBS+ for 60 min at 37 °C. All tissues were blocked in 1% BSA, 1% FBS, DPBS^45^ for one hour prior to undergoing DBTA or immunostaining. Hematoxylin and eosin (H&E) staining of tissue sections was conducted as previously described^65^.

### Dynamic biochemical tissue analysis (DBTA)

Figure 1 shows the experimental set up for this dynamic adhesion assay, which consisted of a parallel plate flow chamber (GlycoTech, Gaithersburg, MD) that was sealed around the tissue. The laminar flow conditions in the flow channel were controlled by a precision syringe pump (Harvard Apparatus, Holliston, MA). Wall shear stress values, which ranged from 0.12(5) dyne/cm^2^ to 2.0 dyne/cm^2^, were calculated using $\tau =\frac{3Q\mu }{2W{H}^{2}}$, which is described extensively elsewhere^36,37,67,68^. The events in the flow chamber were captured with a PixeLINK (Ottawa, ON, Canada) or Retiga-EXi CCD (QImaging, Surrey, BC, Canada) camera connected to a Leica DMI 6000 inverted microscope (Leica Microsystems, Wetzlar, Germany) and recorded via PixeLINK or StreamPix (Norpix, Inc., Montreal, QC, Canada) imaging software. DBTA images shown in all figures are derived from two consecutive, high-resolution video frames that have been overlaid at 50% transparency to reduce the visibility of non-interacting probes.

### DBTA data analysis

DBTA experiments were designed and conducted for multilevel analysis, with the intent to address repeatability and reproducibility. More specifically, technical replicates were conducted to test the variability of the dynamic assay itself. Because the DBTA method is a nondestructive test that offers reduced sample perturbation compared to SBTA, a single serial section could be tested multiple times and with numerous types of probes. In between different trials, DBTA probes were removed from the system. Multi-section experiments were also conducted, where analysis was performed on three consecutive serial sections (technical replicates) from the same sample to study the variability between sections. More importantly, this study contains comprehensive technical replicate analysis coupled with the testing of numerous biological replicates from several distinct cancer cases.

DBTA probe adhesion to the tissue surface was quantified by analyzing DBTA video captured at 10 frames per second in ImageJ^69^ with the assistance of the MOSAIC ParticleTracker^70^ and MTrackJ^71^ programs. The adhesion values reported here represent total adhesion, which includes rolling, transient, and firm adhesion.

For more in-depth characterization of the DBTA probe rolling on the tissue surface, pause time distribution analysis was performed^18–21,23,48,55,72–74^. In order to be classified as rolling, the probes were required to display a velocity that was, at a minimum, an order of magnitude smaller than the velocity of the non-interacting control probes that were in the layer of fluid adjacent to the tissue surface (i.e., in focus). High-speed video microscopy captured through a 40x objective at 175 frames per second with the PixeLINK system detailed above was used to discretize rolling into a sequence of succinct pauses, and these pauses were used to estimate the receptor-ligand dissociation constant, k_off_^48^. Uz and Laurenzi have derived a point estimate for k_off_ based on the method of maximum likelihood and stochastic kinetics^48^. Probe motion and pause duration were calculated using an open source, cross-correlation tracking program^75^. Using the spatiotemporal data from this program, each probe’s velocity profile was calculated over the duration of rolling interaction. A pause was defined as a change in position with respect to time (i.e., velocity) that is equal to zero. For the purposes of this document, and in order to account for spatial and temporal limitations of the measurement system, a pause was more precisely defined as the opposite of motion, which was defined as a change in position that is greater than 1 pixel, or ~0.7 micrometers. For each level of defined wall shear stress, a minimum of 30 separate pauses were measured.

### Immunostaining

The immunofluorescence protocol was a modified version of procedures previously described^45,76^. All blocking and immunostaining (SBTA) steps were performed in a humidified container on a rocker platform. Serial tissue sections were blocked in 1% BSA,1% FBS, DPBS+ (blocking buffer) for one hour then stained with selectin chimera, primary antibody, or appropriate species isotype control at 10 μg/mL. Tissues were then washed in 0.05% Tween-20/DPBS+ and incubated with secondary antibodies at 5 μg/mL in blocking buffer for one hour at RT. Unless indicated, secondary antibodies used were conjugated with near-infrared fluorochromes (e.g., Alexa Fluor 647) in order to avoid signal contamination with tissue autofluorescence. Following, tissues were washed in 0.05% Tween-20/DPBS+ and DPBS+, respectively. In preparation for imaging, all tissues were mounted in ProLong Gold anti-fade reagent and imaged through a 10x objective under widefield fluorescence using a Leica DMI 6000 inverted microscope equipped with an automated filter cube wheel containing the appropriate excitation and emission filters. Images were captured with a Retiga EXi CCD camera and StreamPix imaging software. Background (autofluorescence + isotype) signal of the labeled sample was accounted for by setting the exposure time of the isotype sample to minimize the signal detected, and then using this value as the exposure time for the experimental sample.

### Statistical analysis

For DBTA probe adhesion, an unpaired, two-tailed student’s t-test with an alpha value equal to 0.05 was used. For pause time distribution analysis, both normal and non-normal statistical methods were used in redundancy. Under the presumption the distributions are normal, data shown are mean pause duration in seconds ± SD of 30 separate pauses (n = 30) in a 100 μm × 100 μm region of signet ring cell carcinoma tissue. ANOVA, which is intended for normal distributions but is capable of tolerating data that are non-normal, was used to determine the effect of wall shear stress on pause time duration. Under the presumption the distributions are non-normal, data shown represent the estimation of P-selectin DBTA probe k_off_ using the method of maximum likelihood^48^ from 30 separate pauses (n = 30). To deal with non-normal distributions, Levene’s test and the Mann-Whitney U test were used as a more tolerant version of ANOVA and Student’s t-test, respectively. Error bars are defined in each figure legend.

## Supplementary information

Supplementary Info

Video S1

Video S2

Video S3

Video S4

Video S5

Video S6

Video S7

Video S8

Video S9

Video S10

Video S11

Video S12

Video S13

Video S14

Video S15

Video S16

Video S17

## Data Availability

All relevant data are within the paper and its Supplemental Information files.
