# Supplementary material for: Dynamic biochemical tissue analysis detects functional selectin ligands on human cancer tissues
Source: Sci Rep. 2019 Jun 11;9:8511. doi: 10.1038/s41598-019-44838-4 (PMC6560120; doi:10.1038/s41598-019-44838-4)
Supplement: Supplementary file 1 — Supplementary Info [file 41598_2019_44838_MOESM1_ESM.pdf]

Supplemental Information

Dynamic biochemical tissue analysis detects functional selectin ligands on human cancer tissues

Eric W. Martin[1,2], Ramiro Malgor[1,3], Vicente A. Resto[5], Douglas J. Goetz[1,2], and Monica M. Burdick[1,2,4,*]

[1]Biomedical Engineering Program, [2]Department of Chemical and Biomolecular Engineering, Russ College of Engineering and Technology, [3]Department of Biomedical Sciences, Heritage College of Osteopathic Medicine, and [4]Edison Biotechnology Institute, Ohio University, Athens, OH 45701. [5]Department of Otolaryngology, University of Texas-Medical Branch, Galveston, TX 77555.

*Corresponding author email address: burdick@ohio.edu

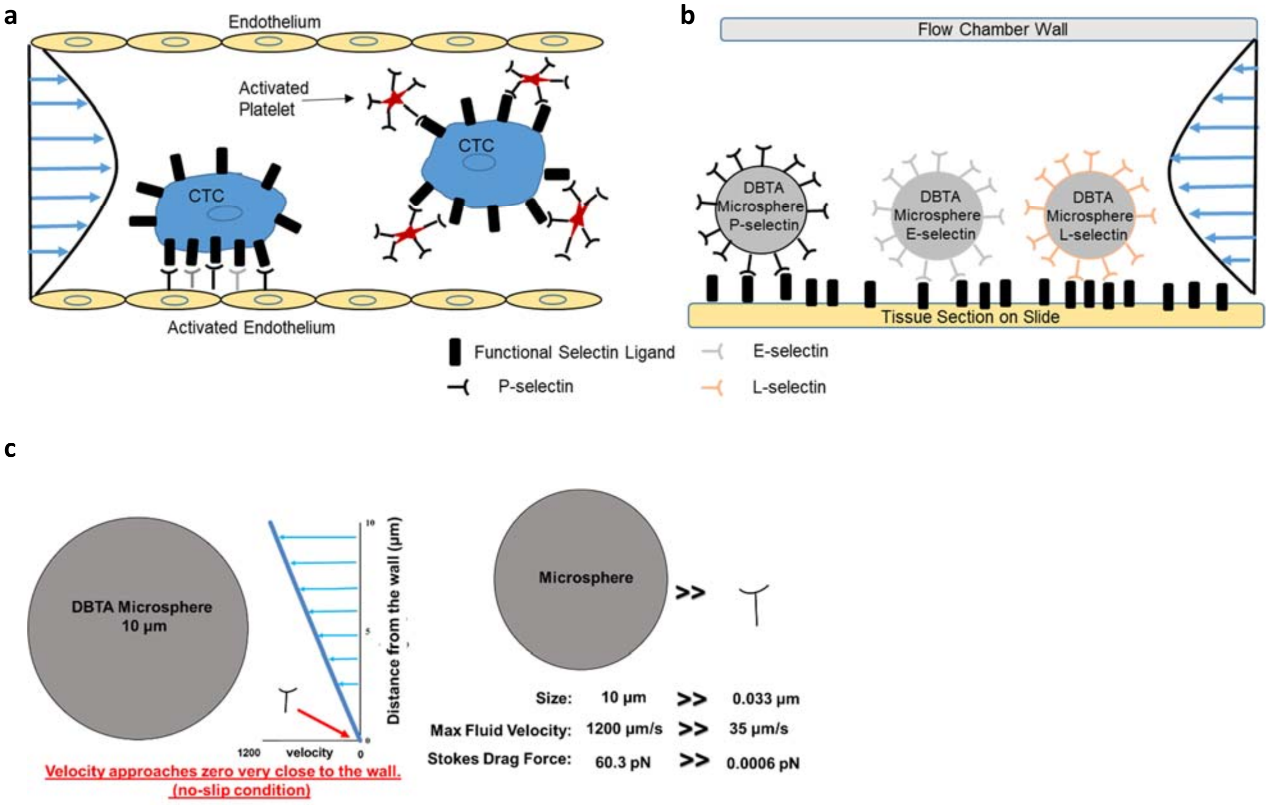

Supplementary Figure S1. Introduction to DBTA (dynamic biochemical tissue analysis) **(a)** Tumor cells may express functional selectin ligands that can bind to selectins expressed by activated platelets (P-selectin), activated endothelium (E- and P-selectin), or leukocytes (L-selectin, not pictured). The cell-cell adhesion mediated by selectin ligands on tumor cells to selectins on the non-tumor cells may facilitate metastatic colonization of secondary sites. **(b)** DBTA involves the perfusion of polystyrene microspheres that are coated with molecular probes (e.g., selectins) at well-defined wall shear stresses. The fluid profile and delivery trajectories of particles in the flow chamber are well-documented[36,37,68,69]. The purpose of coating a particle with the desired molecular probe is to assist in transducing hydrodynamic forces to the desired level of mechanical force that is to be exerted on the bond between the receptor (i.e., the selectin on the probe) and ligand (i.e., the selectin ligand expressed on the tissue section on the slide). As demonstrated in Figures 2-8 of the main manuscript, selectin ligands are detected more often on cancerous tissues than on non-cancerous tissues. Ultimately, detection of selectin ligands *in situ* in tissue sections from tumors may give insights towards the expression and regulation of selectin ligands on circulating tumor cells in (a). **(c)** An order of magnitude analysis shows the viscous drag (force) exerted on the immersed, ideal body of an antibody perfused over a biospecimen at 1.00 dyne/cm^2 is negligible when compared to that of a DBTA probe (microsphere).

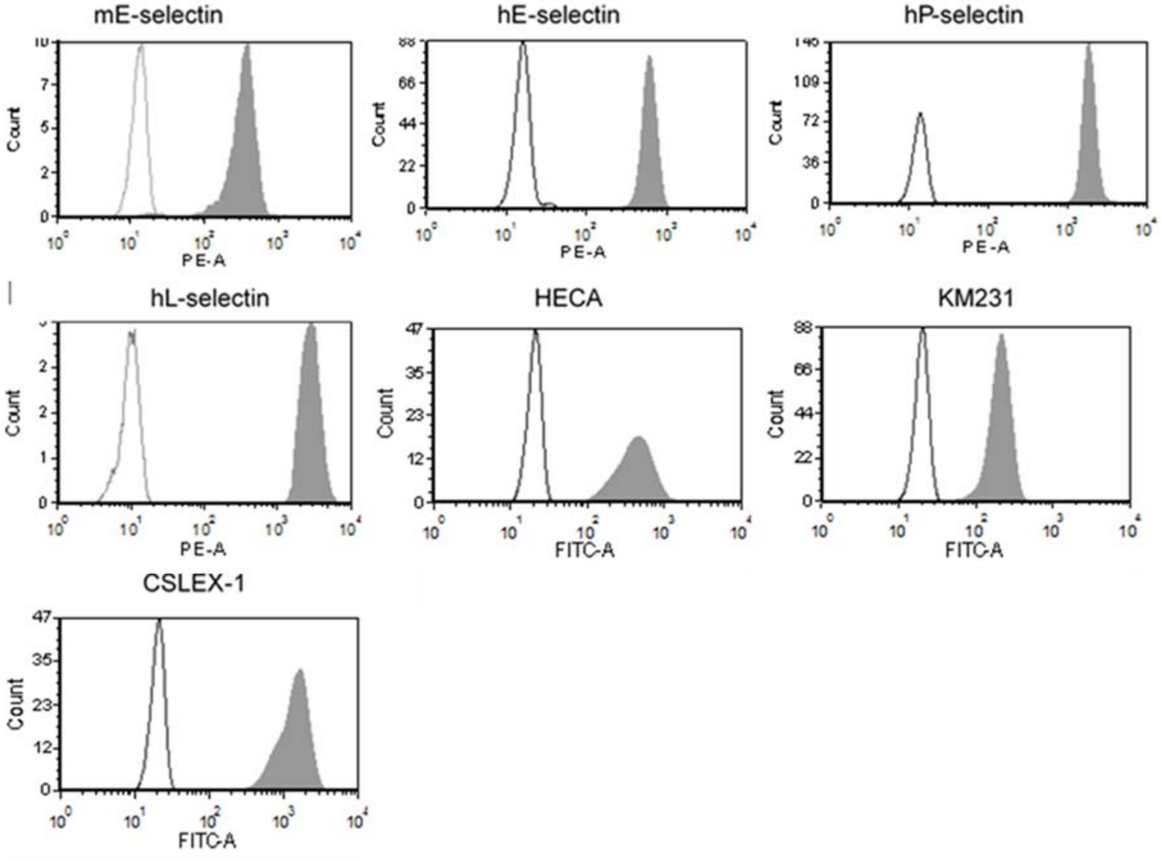

Supplementary Figure S2. Surface coverage of the DBTA probes (polystyrene microspheres conjugated with the indicated molecular probe). For all histograms, filled curves show a clear shift to the right when compared to the control, shown as an open curve, demonstrating successful DBTA probe functionalization.

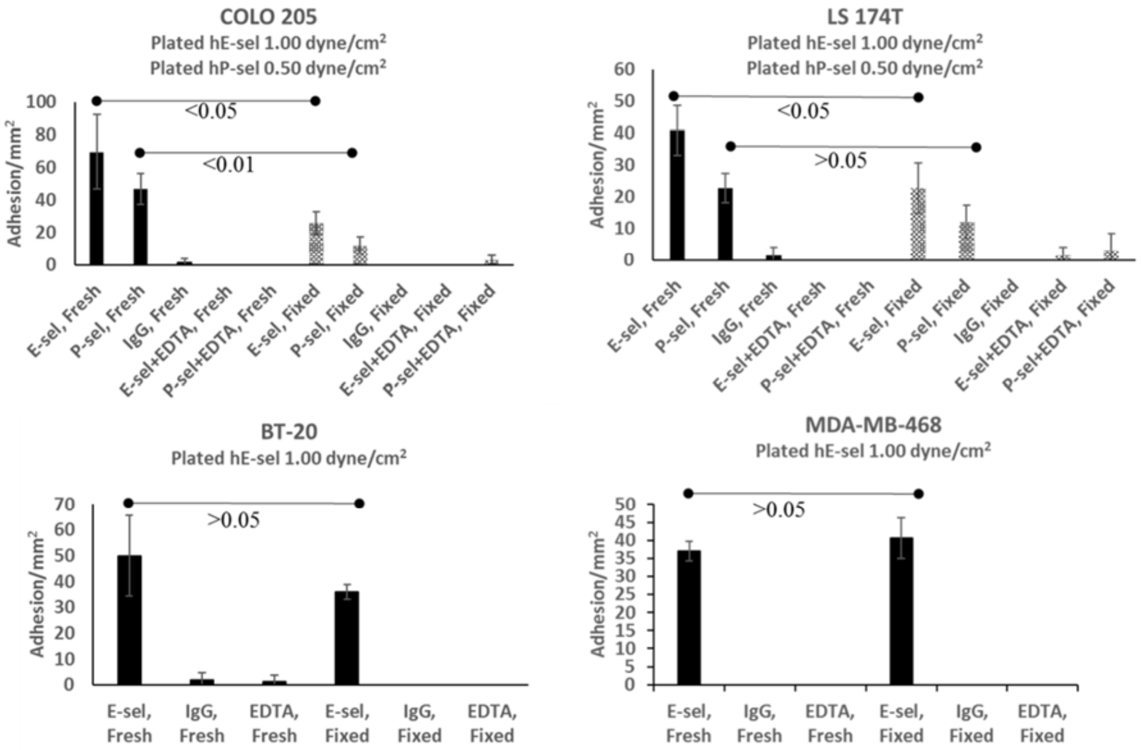

Supplementary Figure S3 Part A. Formalin fixation does not increase binding of cancer cell lines to plated selectins under flow. Untreated (fresh) and formalin-fixed (10,000 cells/mL of 10% neutral buffered formalin for 10 minutes at RT) COLO 205, LS174T, BT-20,and MDA-MB-468 cell lines were perfused over plated (human) E-selectin (5 ug/mL), P-selectin (10 ug/mL), and hIgG (10 ug/mL) at 250,000 cells/mL DPBS+ for 2 minutes at the indicated wall shear stress. Plating occurred overnight at 4°C and was followed by a block in 1% FBS/BSA for 1 hour at 4°C. Cells were harvested with 5 mM EDTA/DPBS-. Prior to perfusion, cell concentration was verified using a Scepter handheld cell counter. The specificity of interaction was confirmed using 10 mM EDTA and plated IgG as negative controls. Data shown are mean adhesion ± SD of three independent experiments. Relevant P-values are shown in the figure and indicate that selectin ligand mediated adhesion is either diminished or unaltered following fixation.

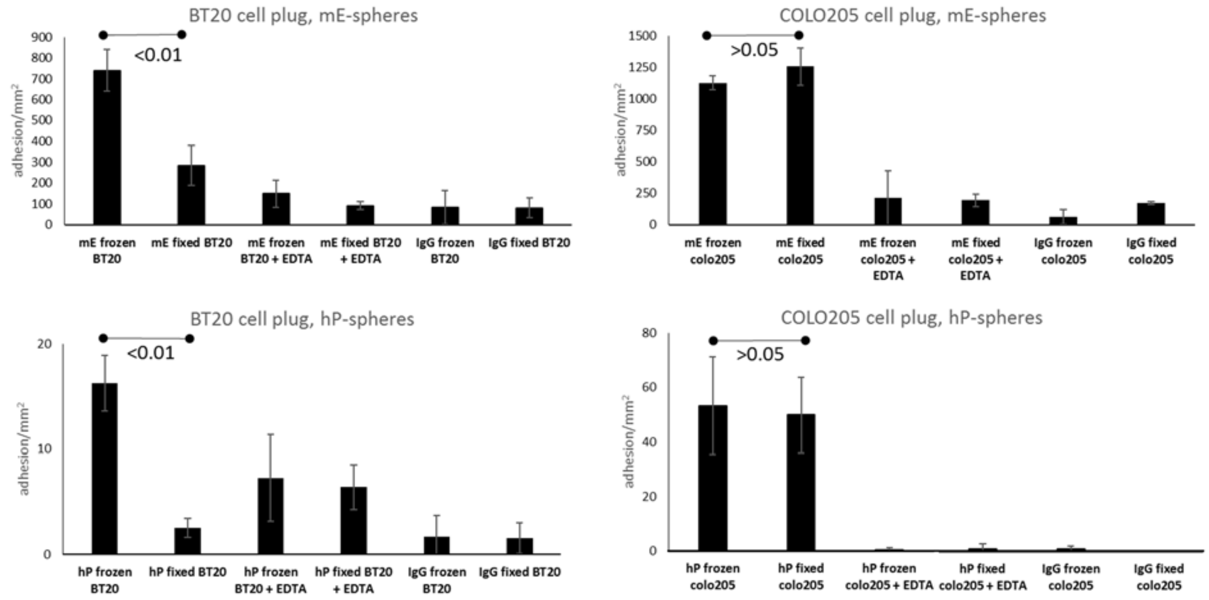

Supplementary Figure S3 Part B. Formalin fixation and paraffin embedding does not increase binding of selectin DBTA probes to mock tissue consisting of microtome-sectioned cell line plugs (pellets). mE- and hP-selectin DBTA probes perfused at 0.50 dyne/cm^2 and 500,000 probes/mL DBPS+ specifically adhered to mock tissues consisting of either BT-20 or COLO 205 microtome-sectioned cell plugs**.** Specificity of interaction was confirmed using 10 mM EDTA and hIgG DBTA probes as negative controls. Data shown are mean adhesion ± SD of three independent experiments in which DBTA probes were perfused over three cell plug serial sections. P-values indicate that selectin ligand mediated adhesion is either diminished or unaltered following formalin fixation and paraffin embedding.

For fresh mock tissue cell line plugs, harvested cells were formed into a pellet using a protocol adapted from the Johns Hopkins University Oncology Tissue Services (which can be found at http://tmalab.jhmi.edu/docs/Cell_line_block_procedure_Jan2013.pdf), embedded in OCT cryomatrix, and sectioned at 5 µm. Then the plug section was placed onto a slide and washed 3x in DPBS and blocked in 1% BSA/1% FBS in DPBS+ for 1 hr prior to conducting DBTA. For mock tissue FFPE cell line plugs, harvested cells were formed into a pellet then fixed in 10% neutral buffered formalin for 24 hours at RT followed by a wash in DPBS x2. The cell pellet was placed in a stainless steel, tissue base mold container and then carefully dehydrated by slowly adding and removing the following liquids in sequence: 70% ethanol, 95% ethanol, 100% ethanol x2, and xylene x3. The cell plug was then embedded in paraffin and sectioned using a microtome at 5 µm. Sections were deparaffinized using consecutive incubations for 3 minutes each in xylene x3, 100% ethanol twice, 95% ethanol, 70% ethanol, and DPBS. Sections of the FFPE cell line plugs were then blocked in 1% BSA and 1% FBS in DPBS+ for 1 hr prior to conducting DBTA.

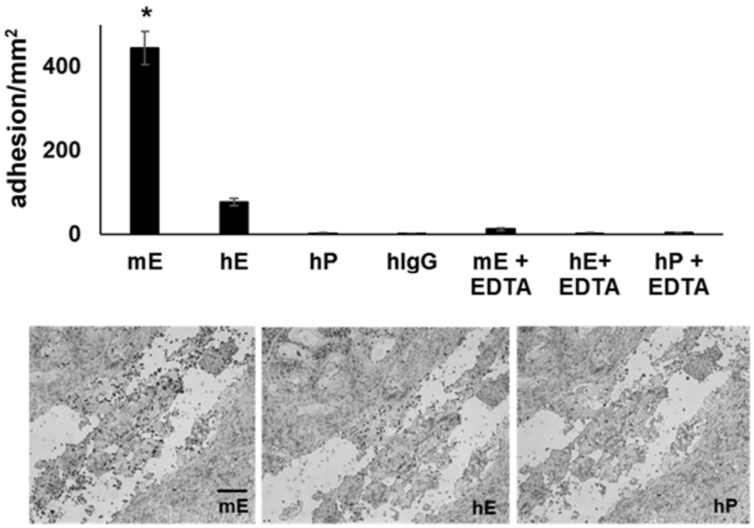

Supplementary Figure S4. The greatest amount of specific adhesion occurs with mE-selectin DBTA probe. mE-, hE-, hP-selectin DBTA probes adhered to lung adenocarcinoma tissue. Probes were perfused at 250,000 probes/mL and 0.50 dyne/cm^2. Data shown are mean adhesion ± SD of three technical replicates and are representative of independent experiments conducted on n> 10 independent cases, including lung, ovarian, pancreatic, and stomach tissue samples. Specificity of interaction was confirmed using hIgG probes as well as with each respective selectin DBTA probe perfused in 10 mM EDTA. Scale bar = 100 μm. * P < 0.001 compared to all other conditions.

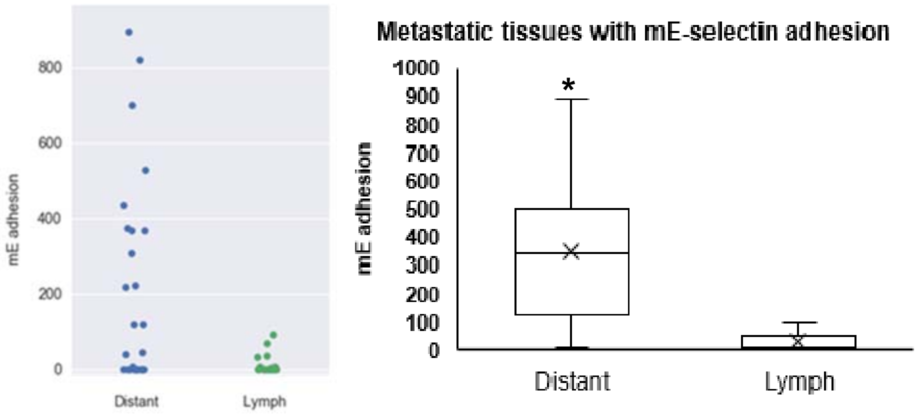

Supplementary Figure S5. mE-selectin DBTA probe adhesion per mm^2 by the site of metastasis, classified as either distant (e.g., hematogenous) or lymphatic.* P = 0.0021.

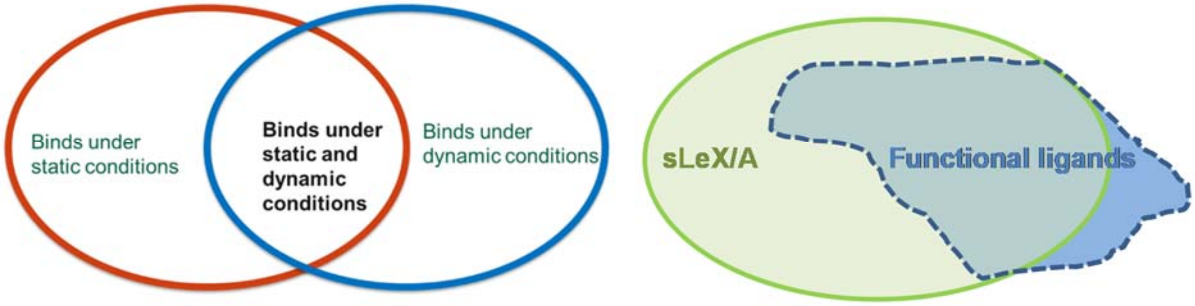

Supplementary Figure S6. Proposed Venn diagrams representing the complex relationship between selectin ligands that bind to selectins under static conditions versus those that bind to selectins under dynamic conditions. This representation extends to the partitioning of functional selectin ligands that express sLeX/A.

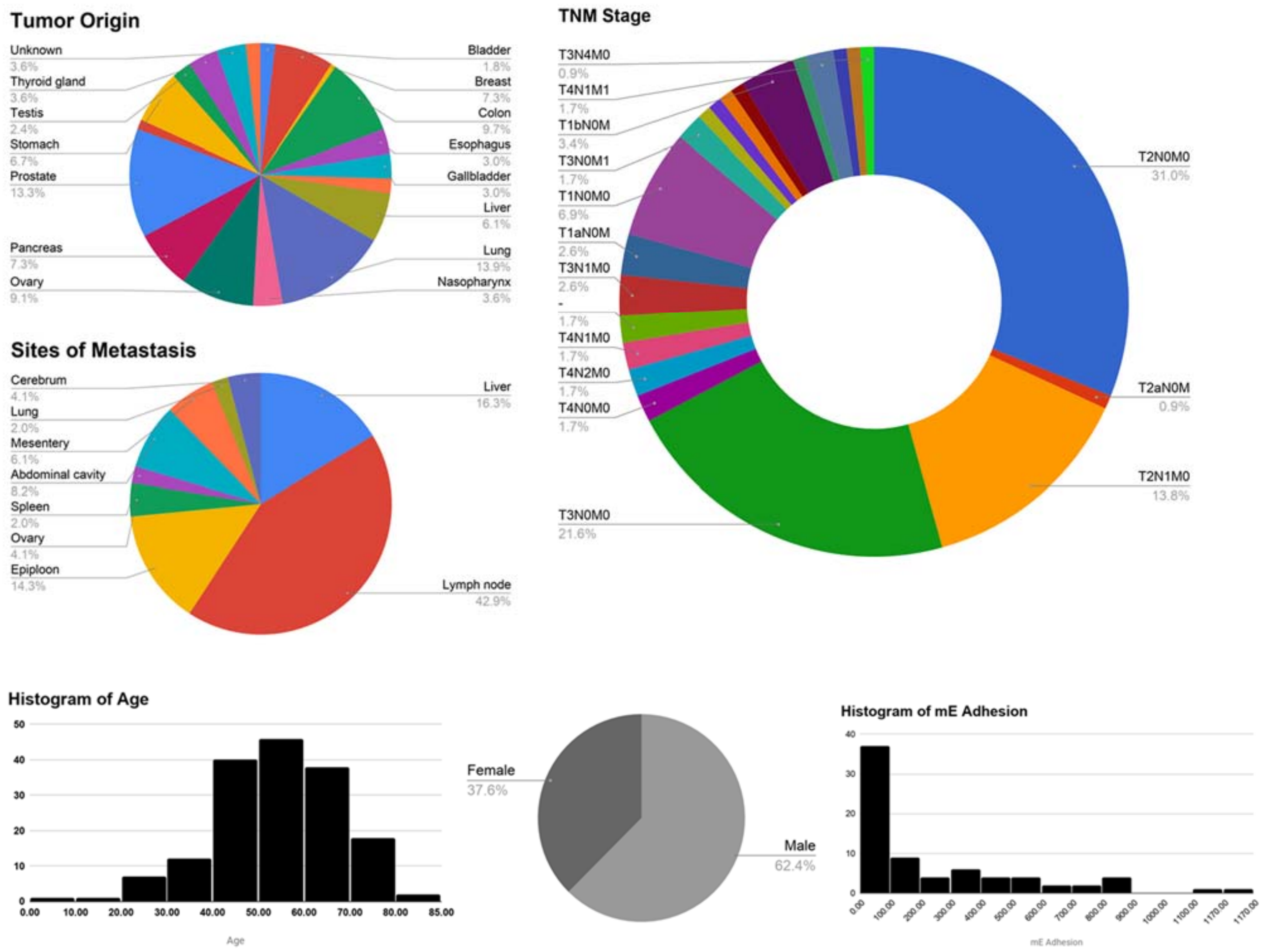

Supplementary Figure S7. Profile of the cases examined in this study.

**Supplementary Table S1.** Corresponding P-values for Figure 6.			
	hP, EDTA	hIgG	hP, sialidase
colon mucinous adenocarcinoma	0.001	0.0008	0.0008
colon adenocarcinoma	0.0036	0.001	0.0017
colon signet ring cell carcinoma	0.0002	<0.0001	0.0001
lung papillary adenocarcinoma	0.0014	0.0005	0.0005
lung bronchioalveolar carcinoma	<0.0001	<0.0001	<0.0001
lung adenocarcinoma	0.0004	<0.0001	<0.0001
ovarian mucinous adenocarcinoma	0.001	0.001	0.001
ovarian endometrioid adenocarcinoma	0.0002	<0.0001	0.0002
pancreatic adenocarcinoma	0.001	<0.0001	0.0009
pancreatic duct adenocarcinoma I	0.022	0.0024	0.01
pancreatic duct adenocarcinoma II	0.0008	<0.0001	<0.0001
stomach adenocarcinoma I	0.011	0.0001	0.046
stomach adenocarcinoma II	0.0002	<0.0001	0.07

Supplementary Table S2

Age	M/F	Organ	Cancer Type	TNM	Grade	met/primary	Met Site	mE adhesion
50	M	Bladder	transitional cell carcinoma	T2N0M0	II	primary		50
68	M	Bladder	transitional cell carcinoma	T2N0M0	II	primary		0
57	M	Bladder	transitional cell carcinoma	T2aN0M0	II	primary		0
34	F	Breast	metastatic adenocarcinoma from breast			metastatic	Liver	0
48	F	Breast	metastatic adenocarcinoma of oxter from breast			metastatic	Lymph node	0
53	F	Breast	metastatic adenocarcinoma of oxter from breast			metastatic	Lymph node	0
66	M	Breast	metastatic adenocarcinoma of oxter from breast			metastatic	Lymph node	0
42	F	Breast	metastatic adenocarcinoma of oxter from breast			metastatic	Lymph node	5
58	F	Breast	metastatic adenocarcinoma of oxter from breast			metastatic	Lymph node	33
46	F	Breast	metastatic adenocarcinoma of oxter from breast			metastatic	Lymph node	69
57	F	Breast	ductal carcinoma	T2N0M0	III	primary		43
50	F	Breast	ductal carcinoma	T2N1M0		primary		168
42	F	Breast	invasive ductal carcinoma	T3N0M0	IIB	primary		0
37	F	Breast	invasive ductal carcinoma	T2N1M0	IIB	primary		0
51	F	Breast	invasive ductal carcinoma	T2N1M0	IIB	primary		0
36	F	Cervix	metastatic squamous cell carcinoma of pelvic cavity from cervix			metastatic	Lymph node	7
67	F	Colon	metastatic adenocarcinoma from colon			metastatic	Epiploon	0
55	M	Colon	metastatic mucinous adenocarcinoma from colon			metastatic	Liver	0
58	M	Colon	metastatic adenocarcinoma from colon			metastatic	Liver	0
54	F	Colon	metastatic mucinous adenocarcinoma from colon			metastatic	Ovary	0
55	M	Colon	metastatic mucinous adenocarcinoma from colon			metastatic	Liver	7
67	F	Colon	metastatic mucinous adenocarcinoma from colon			metastatic	Ovary	374
58	M	Colon	metastatic adenocarcinoma from colon			metastatic	Liver	700
58	F	Colon	metastatic adenocarcinoma from colon			metastatic	Liver	820
57	M	Colon	metastatic adenocarcinoma from colon			metastatic	Epiploon	894
60	M	Colon	adenocarcinoma	T4N0M0	IIBI	primary		141
45	M	Colon	mucinous adenocarcinoma	T4N2M0	III	primary		340
37	F	Colon	signet ring cell carcinoma	T3N0M0	IIA	primary		392
45	M	Colon	mucinous adenocarcinoma	T4N2M0	III	primary		441
48	M	Colon	signet-ring cell carcinoma	T4N1M0	II	primary		548
72	F	Colon	adenocarcinoma	T4N0M0	II	primary		578
22	M	Colon	signet ring cell carcinoma	T4N1M0	III	primary		1137
65	M	Esophagus	squamous cell carcinoma	-	II	primary		166
60	M	Esophagus	adenocarcinoma	T3N1M0	III	primary		171
59	M	Esophagus	squamous cell carcinoma	T3N0M0	IIA	primary		0
50	M	Esophagus	squamous cell carcinoma	T3N0M0	IIA	primary		0
61	M	Esophagus	squamous cell carcinoma	T2N0M0	IIA	primary		0
53	F	Gallbladder	adenocarcinoma	T3N0M0	IIA	primary		151
70	F	Gallbladder	adenocarcinoma	T1aN0M0	I	primary		0
68	M	Gallbladder	adenocarcinoma	T2N0M0	II	primary		0
66	M	Gallbladder	adenocarcinoma	T2N0M0	II	primary		0
54	M	Gallbladder	adenocarcinoma	T2N1M0	III	primary		0
54	M	Kidney	clear cell carcinoma	T1N0M0	I	primary		0
30	F	Kidney	clear cell carcinoma	T3N0M1	IV	primary		0
70	M	Kidney	clear cell carcinoma	T2N1M0	III	primary		0
50	M	Liver	metastatic adenocarcinoma from liver			metastatic	Spleen	119
56	M	Liver	hepatocellular carcinoma	T2N0M0	II	primary		19
57	M	Liver	cholangiocellular carcinoma	T3N0M0	IIIA	primary		42
64	M	Liver	hepatocellular carcinoma	T2N0M0	II	primary		0
49	M	Liver	hepatocellular carcinoma	T2N0M0	II	primary		0
24	M	Liver	sarcomatous hepatocellular carcinoma	T3N0M0	IIIA	primary		0
60	M	Liver	cholangiocellular carcinoma	T3N0M0	IIIA	primary		0
49	M	Liver	clear cell carcinoma	T2N0M0	II	primary		0
46	M	Liver	malignant fibrohistiocytoma	T2N0M0 G1	Ib	primary		0
55	M	Liver	angiosarcoma	T2N0M0 G3	IIIB	primary		0
76	M	Lung	metastatic squamous cell carcinoma of abdominal wall			metastatic	Abdominal cavity	0
48	F	Lung	metastatic squamous cell carcinoma of neck from lung			metastatic	Lymph node	0
43	F	Lung	metastatic adenocarcinoma of neck from lung			metastatic	Lymph node	0
52	M	Lung	metastatic squamous cell carcinoma of oxter from lung			metastatic	Lymph node	36
53	M	Lung	adenosquamous carcinoma	T2N1M0	II	primary		10
61	M	Lung	squamous cell carcinoma	T2N1M0	II	primary		13
49	M	Lung	adenocarcinoma	T2N0M0	I	primary		19
51	M	Lung	squamous cell carcinoma	T3N0M0	IIIa	primary		29
48	M	Lung	large cell carcinoma	T3N3M0	IIIb	primary		45
22	F	Lung	adenocarcinoma	T2N0M0	I	primary		89
56	F	Lung	papillary adenocarcinoma	T2N0M0	I	primary		230
43	F	Lung	adenocarcinoma	T2N2M0	IIA	primary		483
35	M	Lung	atypical carcinoid	T2N1M0	II	primary		490
71	M	Lung	adenocarcinoma	T2N1M0	II	primary		573
41	M	Lung	adenocarcinoma	T2N0M0	I	primary		614
52	M	Lung	bronchioloalveolar carcinoma	T2N1M0	II	primary		796
42	M	Lung	adenocarcinoma	T2N0M0	I	primary		0
67	M	Lung	squamous cell carcinoma	T2N1M0	II	primary		0
70	M	Lung	adenocarcinoma	T2N0M0	I	primary		0
44	M	Lung	small cell undifferentiated carcinoma	T2N1M0	II	primary		0
43	M	Lung	carcinoid	T2N0M0	I	primary		0
57	F	Lung	bronchioloalveolar carcinoma	T2N1M0	II	primary		0
30	F	Lung	large cell carcinoma	T2N1M0	II	primary		0
34	M	Nasopharynx	metastatic squamous cell carcinoma of neck from nasopharynx			metastatic	Lymph node	0
35	M	Nasopharynx	metastatic squamous cell carcinoma of neck from nasopharynx			metastatic	Lymph node	0
49	M	Nasopharynx	metastatic squamous cell carcinoma of neck from nasopharynx			metastatic	Lymph node	0

Supplementary Table S2 continued								
51	M	Nasopharynx	metastatic squamous cell carcinoma of neck from nose			metastatic	Lymph node	0
51	M	Nasopharynx	metastatic squamous cell carcinoma of neck from nasopharynx			metastatic	Lymph node	4
64	F	Nasopharynx	metastatic squamous cell carcinoma of neck from nasopharynx			metastatic	Lymph node	7
64	F	Ovary	metastatic serous papillary adenocarcinoma from ovary			metastatic	Epiploon	0
59	F	Ovary	metastatic mucinous adenocarcinoma from ovary			metastatic	Epiploon	0
43	F	Ovary	metastatic adenocarcinoma from ovary			metastatic	Epiploon	0
59	F	Ovary	metastatic serous adenocarcinoma from ovary			metastatic	Mesentery	0
49	F	Ovary	metastatic serous adenocarcinoma of peritoneum from ovary			metastatic	Abdominal cavity	368
42	F	Ovary	mucinous adenocarcinoma	T1bN0M0	Ib	primary		15
43	F	Ovary	serous papillary adenocarcinoma	T1N0M0	I	primary		32
52	F	Ovary	serous papillary adenocarcinoma	T1bN0M0	Ib	primary		34
41	F	Ovary	mucinous adenocarcinoma	T1aN0M0	Ia	primary		66
49	F	Ovary	clear cell carcinoma	T1N0M0	I	primary		88
26	F	Ovary	serous papillary adenocarcinoma	T1cN0M0	Ic	primary		136
43	F	Ovary	endometrioid adenocarcinoma	T1bN0M0	Ib	primary		1170
37	F	Ovary	clear cell carcinoma	T1aN0M0	Ia	primary		0
40	F	Ovary	endometrioid adenocarcinoma	T2N1M0	IIIc	primary		0
25	F	Ovary	granular cell tumor	T1N0M0	I	primary		0
65	M	Pancreas	metastatic adenocarcinoma from pancreas			metastatic	Mesentery	45
52	M	Pancreas	adenosquamous carcinoma	T3N0M0	II	primary		26
52	M	Pancreas	duct adenocarcinoma	T3N0M0	II	primary		37
44	M	Pancreas	duct adenocarcinoma	T3N0M0	II	primary		42
51	F	Pancreas	neuroendocrine carcinoma	-	-	primary		53
57	M	Pancreas	adenocarcinoma	T3N0M0	II	primary		85
77	F	Pancreas	duct adenocarcinoma	T2N0M0	I	primary		863
56	M	Pancreas	duct adenocarcinoma	T2N1M0	III	primary		0
49	M	Pancreas	adenosquamous carcinoma	T3N0M0	II	primary		0
46	M	Pancreas	pseudopapillary tumor	T2N0M0	I	primary		0
54	F	Pancreas	pseudopapillary tumor	T2N0M0	I	primary		0
68	F	Pancreas	leiomyosarcoma	T2N0M0	Ib	primary		0
60	M	Prostate	undifferentiated carcinoma	T2N0M0	-	primary		32
66	M	Prostate	adenocarcinoma	T2N0M0	II	primary		53
82	M	Prostate	adenocarcinoma	T3N0M0	III	primary		0
76	M	Prostate	adenocarcinoma	T2N0M0	III	primary		0
60	M	Prostate	adenocarcinoma	T4N1M1c	IV	primary		0
64	M	Prostate	adenocarcinoma	T3N0M1	IV	primary		0
80	M	Prostate	adenocarcinoma	T4N1M1c	IV	primary		0
75	M	Prostate	adenocarcinoma	T2N1M1c	IV	primary		0
69	M	Prostate	adenocarcinoma	T2N0M0	II	primary		0
69	M	Prostate	small acinous carcinoma	T2N0M0	-	primary		0
76	M	Prostate	adenocarcinoma	T3N1M1b	-	primary		0
72	M	Prostate	adenocarcinoma	T3N0M0	-	primary		0
64	M	Prostate	adenocarcinoma	T1N0M0	I	primary		0
73	M	Prostate	adenocarcinoma	T2N0M0	II	primary		0
70	M	Prostate	adenocarcinoma	T3N0M0	III	primary		0
71	M	Prostate	adenocarcinoma	T2N0M0	II	primary		0
64	M	Prostate	adenocarcinoma	T2N0M0	II	primary		0
61	M	Prostate	adenocarcinoma	T3N1M0	III	primary		0
65	M	Prostate	adenocarcinoma	T2N0M0	II	primary		0
66	M	Prostate	adenocarcinoma	T3N0M0	III	primary		0
75	M	Prostate	adenocarcinoma	T2N0M0	II	primary		0
72	M	Prostate	leiomyosarcoma	T1N0M0	Ia	primary		0
64	F	Rectum	metastatic adenocarcinoma from rectum			metastatic	Epiploon	40
63	F	Rectum	metastatic adenocarcinoma from rectum			metastatic	Liver	368
70	M	Stomach	metastatic adenocarcinoma of abdominal wall from stomach			metastatic	Abdominal cavity	222
65	M	Stomach	metastatic adenocarcinoma from stomach			metastatic	Lung	435
53	M	Stomach	metastatic adenocarcinoma from stomach			metastatic	Liver	528
50	F	Stomach	adenocarcinoma	T3N0M0	II	primary		68
56	M	Stomach	adenocarcinoma	T3N0M0	II	primary		151
42	M	Stomach	carcinoid	T3N0M0	II	primary		275
66	M	Stomach	adenocarcinoma	T2N0M0	IB	primary		0
69	M	Stomach	adenocarcinoma	T2N0M0	IB	primary		0
50	F	Stomach	adenocarcinoma	T3N0M0	II	primary		0
55	M	Stomach	adenocarcinoma	T3N4M0	IIIb	primary		0
42	M	Stomach	carcinoid	T3N0M0	II	primary		0
1	M	Testis	yolk sac tumor	T2N0M0	I	primary		0
26	M	Testis	seminoma	T2N0M0	I	primary		0
43	M	Testis	seminoma	T1N0M0	I	primary		0
24	M	Testis	embryonal carcinoma	T1N0M0	I	primary		0
45	F	Thyroid gland	metastatic papillary adenocarcinoma of right frontal lobe from thyroid			metastatic	Cerebrum	0
64	M	Thyroid gland	metastatic papillary carcinoma of neck from thyroid			metastatic	Lymph node	0
68	M	Thyroid gland	metastatic papillary carcinoma of neck from thyroid			metastatic	Lymph node	0
49	F	Thyroid gland	metastatic papillary carcinoma of neck from thyroid			metastatic	Lymph node	2
36	F	Thyroid gland	papillary carcinoma	T2N0M0	I	primary		647
19	F	Thyroid gland	papillary carcinoma	T3N0M0	I	primary		875
43	F	Unknown	metastatic serous papillary adenocarcinoma of peritoneum from unknown site			metastatic	Abdominal cavity	0
54	F	Unknown	metastatic papillary adenocarcinoma of left cerebellum from unknown			metastatic	Cerebrum	119
68	M	Unknown	metastatic adenocarcinoma from unknown site			metastatic	Mesentery	218
65	M	Unknown	metastatic mucinous adenocarcinoma from unknown site			metastatic	Epiploon	308
39	M	Unknown	metastatic small cell carcinoma of neck from unknown site			metastatic	Lymph node	0
73	F	Unknown	metastatic squamous cell carcinoma of neck from unknown site			metastatic	Lymph node	92
45	F	Uterine cervix	squamous cell carcinoma	T3N1M0	IIIB	primary		7
49	F	Uterine cervix	squamous cell carcinoma	T1bN0M0	IB	primary		11

**Supplementary Table S3** Corresponding videos for figures in the main text.	
**Video S1**	Fig. 2 and Fig. 4 P-sel SRCC 22 year old
**Video S2**	Fig. 2 hIgG SRCC 22 year old
**Video S3**	Fig. 2 P-sel SRCC 22 year old + EDTA at 15 seconds
**Video S4**	Fig. 2 and Fig. 4 P-sel SRCC 48 year old
**Video S5**	Fig. 2 hIgG SRCC 48 year old
**Video S6**	Fig. 2 P-sel SRCC 48 year old + EDTA at 15 seconds
**Video S7**	Fig. 2 and Fig. 4 P-sel mucinous adenocarcinoma
**Video S8**	Fig. 3 P-sel SRCC 48 year old, high frame rate
**Video S9**	Fig. 4 HECA-452 SRCC 22 year old
**Video S10**	Fig. 4 HECA-452 SRCC 48 year old
**Video S11**	Fig. 5 P-sel adenocarcinoma
**Video S12**	Fig. 6 P-sel ovarian endometrioid adenocarcinoma
**Video S13**	Fig. 6 P-sel ovarian endometrioid adenocarcinoma + sialidase
**Video S14**	Fig. 6 P-sel lung papillary adenocarcinoma
**Video S15**	Fig. 6 P-sel lung papillary adenocarcinoma + sialidase
**Video S16**	Fig. 6 P-sel lung bronchioloalveolar carcinoma
**Video S17**	Fig. 6 P-sel lung bronchioloalveolar carcinoma + sialidase

Video S1 (Fig. 2 and Fig. 4 P-sel SRCC 22 year old)
P-selectin DBTA probes adhered to signet ring cell carcinoma (SRCC) tissue from a 22 year old patient.

Video S2 (Fig. 2 P-sel SRCC 22 year old + EDTA at 15 seconds)
P-selectin DBTA probes adhered to signet ring cell carcinoma (SRCC) tissue from a 48 year old patient was disrupted by the introduction of 10 mM EDTA, a divalent cation chelator that was used to remove Ca^{2+} from the perfusion buffer.

Video S3 (Fig. 2 hIgG SRCC 22 year old)
With respect to P-selectin DBTA probes, significantly fewer human IgG (hIgG) DBTA probes adhered to signet ring cell carcinoma (SRCC) tissue from a 22 year old patient.

Video S4 (Fig. 2 and Fig. 4 P-sel SRCC 48 year old)
P-selectin DBTA probes adhered to colon mucinous adenocarcinoma tissue.

Video S5 (Fig. 2 P-sel SRCC 48 year old + EDTA at 15 seconds)
P-selectin DBTA probes adhered to signet ring cell carcinoma (SRCC) tissue from a 48 year old patient was disrupted by the introduction of 10 mM EDTA, a divalent cation chelator that was used to remove Ca^{2+} from the perfusion buffer. Effects of EDTA can be seen after 15 seconds.

Video S6 (Fig. 2 hIgG SRCC 48 year old)
With respect to P-selectin DBTA probes, significantly fewer human IgG (hIgG) DBTA probes adhered to signet ring cell carcinoma (SRCC) tissue from a 48 year old patient.

Video S7 (Fig. 2 and Fig. 4 P-sel mucinous adenocarcinoma)
P-selectin DBTA probes adhered to colon mucinous adenocarcinoma tissue.

Video S8 (Fig. 3 P-sel SRCC 48 year old, high frame rate)
A representative P-selectin DBTA probe rolling on signet ring cell carcinoma (SRCC) tissue from a 48 year old patient is shown in this video, which was captured through a 40X objective at 175 frames per second.

Video S9 (Fig. 4 HECA-452 SRCC 22 year old)
HECA-452 DBTA probe adhered to signet ring cell carcinoma (SRCC) tissue from a 22 year old patient.

Video S10 (Fig. 4 HECA-452 SRCC 48 year old)
HECA-452 DBTA probe adhered to signet ring cell carcinoma (SRCC) tissue from a 48 year old patient.

Video S11 (Fig. 5 P-sel adenocarcinoma)
P-selectin DBTA probes adhered to colon adenocarcinoma tissue.

Video S12 (Fig. 6 P-sel ovarian endometrioid adenocarcinoma)
P-selectin DBTA probes adhered to ovarian endometrioid adenocarcinoma tissue.

Video S13 (Fig. 6 P-sel ovarian endometrioid adenocarcinoma + sialidase treatment)

Significantly fewer P-selectin DBTA probes adhered to ovarian endometrioid adenocarcinoma tissue following treatment with sialidase.

Video S14 (Fig. 6 P-sel lung papillary adenocarcinoma)
P-selectin DBTA probes adhered to lung papillary adenocarcinoma.

Video S15 (Fig. 6 P-sel lung papillary adenocarcinoma + sialidase treatment)
Significantly fewer P-selectin DBTA probes adhered to lung papillary adenocarcinoma tissue following treatment with sialidase.

Video S16 (Fig. 6 P-sel lung bronchioloalveolar carcinoma)
P-selectin DBTA probes adhered to lung bronchioloalveolar carcinoma.

Video S17 (Fig. 6 P-sel lung bronchioloalveolar carcinoma + sialidase)
Significantly fewer P-selectin DBTA probes adhered to lung bronchioloalveolar carcinoma tissue following treatment with sialidase.
